# Acute effects of a beverage containing bitter melon extract (CARELA) on postprandial glycemia among prediabetic adults

**DOI:** 10.1038/nutd.2016.51

**Published:** 2017-01-16

**Authors:** C H Boone, J R Stout, J A Gordon, M J Redd, D D Church, L P Oliveira, D H Fukuda, J R Hoffman

**Affiliations:** 1 Institute of Exercise Physiology and Wellness, Sport and Exercise Science, University of Central Florida, Orlando, FL, USA; 2 Department of Internal Medicine, College of Medicine, University of Central Florida, Orlando, FL, USA

## Abstract

**Background:**

Acute ingestion of bitter melon (BM) has been shown to suppress the postprandial glycemic response in diabetics, but its impact on glucose regulation among individuals with impaired glucose tolerance is unclear. Moreover, one's glucose tolerance level may influence the effectiveness of BM. This study aimed to examine the acute effects of a beverage containing BM extract on blood glucose regulation during an oral glucose tolerance test (OGTT) among prediabetics.

**Methods:**

Ten prediabetic adults completed two OGTTs—glucose only (D2) and glucose+BM (D3). Responders were identified as subjects whose area under the glucose curve (AUC_glu_) during D3 was lower than D2. To compare the acute effects of the beverage among individuals with varying glucose tolerance levels, subjects were grouped by their glucose response pattern—Fast_peak_ (peak glucose (Glu_peak_) at 30 min postglucose (30P)) and Slow_peak_ (Glu_peak_ after 30P).

**Results:**

During D3, responders (*n*=5) experienced a 13.2% reduction in AUC_glu_ (95% confidence interval (CI): −18.1% to −8.3%), 12.2% reduction in mean glucose (95% CI: −17.3% to −7.0%) and 10.6% reduction in Glu_peak_ (95% CI: −17.5% to −3.7%); plasma glucose was reduced by 9.1% at 30P (95% CI: −15.6% to −2.6%), −24.0% at 60P (95% CI: −36.8% to −11.2%) and −20.0% at 90P (95% CI: −35.8% to −4.2%) during D3. No between-trial differences were noted for Fast_peak_ or Slow_peak_.

**Conclusions:**

Acute ingestion of BM prior to the second OGTT (D3) led to a reduced postprandial glucose response in 50% of the subjects but did not affect the insulin response. Furthermore, the effectiveness of the beverage was seemingly uninfluenced by the subjects' glucose tolerance level. Although BM has shown to aid blood glucose management in diabetics, it remains uncertain why only a portion of subjects responded positively to the BM extract in the current study.

## Introduction

*Momordica charantia*, also known as bitter melon (BM), has been purported to have an antihyperglycemic effect, which may serve as a treatment option for managing diabetes mellitus.^1, 2, 3, 4^ Further, the ingestion of BM has been shown to elicit an acute hypoglycemic effect^5, 6, 7, 8^ and improve the postprandial glycemic response during oral glucose tolerance testing among diabetics.^6, 9, 10^ Conversely, others have reported no significant reduction in postprandial blood glucose concentrations during glucose tolerance testing among non-diabetics,^11, 12^ highlighting the notion that one's ability to maintain blood glucose homeostasis may determine the efficacy of BM. In support, previous studies have shown the presence of responders and non-responders to BM ingestion.^9, 10, 11, 13^

Although there have been a number of studies examining the antidiabetic properties of BM in both animals and humans, minimal research has examined these effects with regard to prediabetics. In fact, evidence of the effects of BM on glucose regulation in prediabetics has merely been inferred from studies among diabetics.^14^ Individuals with prediabetes suffer from moderate glucose dysregulation via impaired glucose tolerance and/or impaired fasting glucose^15^ and are at risk for developing type 2 diabetes mellitus if left untreated.^16^ Moreover, persistently elevated blood glucose levels are associated with many other comorbidities, such as kidney disease, neuropathies and macrovascular complications.^16^ Nevertheless, it is possible for one to convert to a healthy, normoglycemic state through certain lifestyle, pharmacological and/or nutritional interventions^14, 16^; thus the ingestion of an antihyperglycemic agent such as BM may help to minimize the risk of disease progression. Therefore, the purpose of this investigation was to examine the efficacy of BM extract to aid blood glucose regulation in a group of prediabetic adults.

## Materials and methods

### Subjects

Ten prediabetic adults with fasting plasma glucose concentrations between 99 and 126 mg dl^−1^ and/or hemoglobin A1c level between 5.7% and 6.4% volunteered to participate in this single-blinded, cross-over design investigation. Prior to data collection, all subjects completed confidential health questionnaires to assess health status and possible risk factors. Additionally, all subjects provided written consent prior to any data collection. Subjects were asked to avoid participation in any other clinical/investigational trials throughout the duration of this experiment. The New England Independent Review Board's approval was obtained prior to data collection.

### Experimental trials

All subjects were asked to visit the University's Human Performance Laboratory on three separate occasions. During the first visit (D1), laboratory staff provided a thorough explanation of all procedures, expectations and potential risks of study participation; determined subject eligibility; and assessed body composition via air displacement plethysmography (BodPod, COSMED, Chicago, IL, USA). On the second visit (D2), subjects reported to the laboratory following an 8–10-h fast and underwent an oral glucose tolerance test (OGTT). Following a minimum of 96 h, subjects reported to the laboratory (D3) following an 8–10-h fast and underwent an additional OGTT. During D3, subjects consumed a beverage containing 1.25–3 g of BM extract 30 min prior to the OGTT.

During each experimental trial, subjects were asked to ingest a 75-g glucose beverage (SUN-DEX, Fisher Healthcare, Houston, TX, USA) within a 5-min period. For the duration of the standard 2-h OGTT, blood samples were collected every 30 min.

### Blood measurements

During each experimental trial, blood samples were collected at six time points. During D2, blood samples were collected upon arrival (BL); after 30 min, another sample was taken immediately prior to glucose intake (PRE), then again 30, 60, 90 and 120 min after glucose intake (30P, 60P, 90P and 120P, respectively). During D3, the BM beverage was consumed promptly after the BL blood draw; and all subsequent blood draws followed the same protocol as D2. In between blood draws, participants remained inactive (that is, seated or lying down) in the Human Performance Laboratory. All blood draws were obtained following a 15-min equilibration period wherein subjects were instructed to lie in a supine position. All blood samples were obtained using a Teflon cannula placed in a superficial forearm vein using a three-way stopcock with a male luer-lock adapter and plastic syringe. The cannula was maintained patent using an isotonic saline solution (Becton Dickinson and Co., Franklin Lakes, NJ, USA).

At each time point, blood samples were collected into two 6 ml Vacutainer tubes—one untreated and the other coated with K_2_EDTA (Becton Dickinson and Co., Franklin Lakes, NJ). Samples were subsequently centrifuged at 3000 r.p.m. for 15 min at 4 °C. The resulting supernatants were aliquoted into separate micro-centrifuge tubes and frozen at −80 °C for later analyses.

### Biochemical analyses

Plasma glucose was assessed using the glucose oxidase method via an automated analyzer (Analox GM7, Analox Instruments Ltd., Lunenburg, MA, USA). Serum insulin concentrations were determined via commercially available enzyme-linked immunosorbent assay kits (IS130D; Calbiotech, Inc., Spring Valley, CA, USA) and a spectrophotometer (Eon, BioTek Instruments, Inc., Winooski, VT, USA). Following analyses, total area under the glucose and insulin curves (AUC_glu_ and AUC_ins_, respectively) were calculated using a standard trapezoidal method. To eliminate interassay variance, all samples were analyzed in duplicate by a single technician. The coefficient of variation for each assay was 0.87% for glucose and 8.24% for insulin.

### Grouping

Subjects were grouped according to their distinct response to the initial OGTT based on previously published methods;^17, 18^ subjects were categorized by their postprandial glucose response pattern—Fast_peak_ (peak glucose concentration observed at 30P) and Slow_peak_ (peak glucose concentration observed after 30P).

### Statistical analyses

#### All participants

Acute effects of the study beverage on biochemical measures among all prediabetic subjects were analyzed using separate two-way, repeated-measures (trial (D2 vs D3) × time (BL vs PRE vs 30P vs 60P vs 90P vs 120P)) analyses of variance (ANOVAs).

#### Responders

Previous research suggests that BM may lower postprandial glucose in a portion of its consumers.^9, 10, 11, 13^ Therefore, further analyses examined the effects of the study beverage among subjects who were determined to be responders—defined as individuals whose total AUC_glu_ during D3 was lower than D2. Separate three-way, repeated-measures (group (responders vs non-responders) × trial (D2 vs D3) × time (BL vs PRE vs 30P vs 60P vs 90P vs 120P)) ANOVAs were used to assess the acute effects of the study beverage on the postprandial glucose and insulin responses among responders.

#### Fast_peak_ vs Slow_peak_

Acute effects of the study beverage on biochemical measures among subjects with distinct glucose responses were analyzed using separate three-way, repeated-measures (group (Fast_peak_ vs Slow_peak_) × (trial (D2 vs D3) × time (BL vs PRE vs 30P vs 60P vs 90P vs 120P)) ANOVAs.

In the event of significant interactions, least significant difference *post hoc* tests were used for pairwise comparisons. To compare between-trial differences among single-time point measures (for example, AUC, mean and peak concentrations), paired-samples *t*-tests were used. Results were considered significant at an alpha level of *P*⩽0.05, while trends toward significance were acknowledged at *P*⩽0.10. Data were analyzed via IBM SPSS Statistics for Windows (version 23.0, IBM Inc., Armonk, NY, USA). All data are reported as mean±s.d.

## Results

Subject characteristics are displayed in Table 1. Percentage of body fat and body mass index were not significantly different between Fast_peak_ and Slow_peak_ (*P*=0.416 and *P*=0.437, respectively), but Fast_peak_ tended to be younger than Slow_peak_ (95% CI: 45.6–66.9 vs 57.7–72.8; *P*=0.093). When comparing responders and non-responders, no significant differences were noted for percentage of body fat (*P*=0.491), body mass index (*P*=0.948) or age (*P*=0.143).

### Changes in the postprandial glycemic response

Glycemic responses during each experimental trial are displayed in Table 2. When assessing all subjects, no significant trial × time interaction was observed for plasma glucose concentrations (*P*=0.732), but significant time effects were noted (*P*<0.001). Plasma glucose was significantly elevated from BL (95% CI: 100.0–115.1) at 30P (95% CI: 143.9–174.5; *P*<0.001), 60P (95% CI: 119.6–174.5; *P*=0.005) and 90P (95% CI: 110.6–161.8; *P*=0.023). No significant between-trial differences were observed for AUC_glu_ (*P*=0.723), mean plasma glucose (Glu_mean_; *P*=0.720) or peak plasma glucose (Glu_peak_; *P*=0.939).

**Figure 1:**
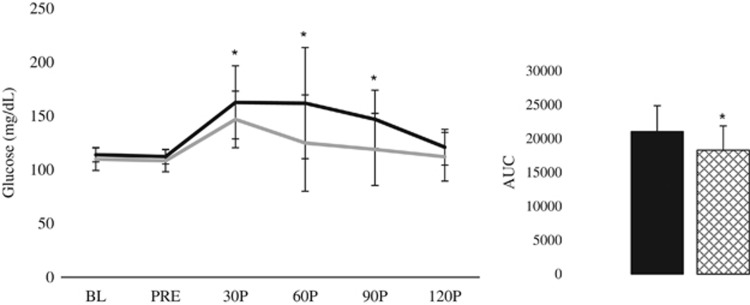
Postprandial glycemic response among responders during an OGTT with (D3; gray line) and without (D2; black line) the acute BM extract ingestion. Inset: net AUC_glu_—D2 is represented by a black bar; D3 is represented by crosshatched bar. *Significantly different from D2.

When assessing the effects of the BM beverage among responders (*n*=5) and non-responders (*n*=5), a significant group × trial × time interaction was observed for plasma glucose concentrations (*P*=0.008; Figure 1). A significant trial × time interaction was observed for responders (*P*=0.004) but not for non-responders (*P*=0.144). Additionally, no significant group × time interactions were noted during either trial (D2: *P*=0.813; D3: *P*=0.149); but significant time effects were observed (*P*=0.001). Plasma glucose concentrations were elevated from BL (95% CI: 100.2–114.9) at 30P (95% CI: 142.9–175.4; *P*<0.001), 60P (95% CI: 117.5–176.6; *P*=0.007) and 90P (95% CI: 108.6–163.8; *P*=0.029). Furthermore, significant group × trial interactions were noted at 30P (*P*=0.002), 60P (*P*=0.003) and 90P (*P*=0.002). Compared with D2, responders displayed significantly lower plasma glucose concentrations at 30P (95% CI: 120.5–204.8 vs 114.3–179.4; *P*=0.027), 60P (95% CI: 97.8–226.1 vs 69.3–180.5; *P*=0.027) and 90P (95% CI: 113.4–180.5 vs 77.3–160.6; *P*=0.027) during D3.

In comparison to D2, responders displayed significantly lower AUC_glu_ (95% CI: 16 284–25 797 vs 13 875–22 729; *P*=0.002), Glu_mean_ (95% CI: 107.9–165.0 vs 92.3–148.0; *P*=0.002) and Glu_peak_ (95% CI: 123.0–214.9 vs 116.6–182.5; *P*=0.020). When assessing non-responders, AUC_ins_ and mean serum insulin (Ins_mean_) during D3 tended to be higher than D2 (*P*=0.064 and *P*=0.095, respectively) while peak serum insulin (Ins_peak_) was significantly greater (95% CI: 18.7–57.1 vs 11.1–40.4; *P*=0.049).

**Figure 2:**
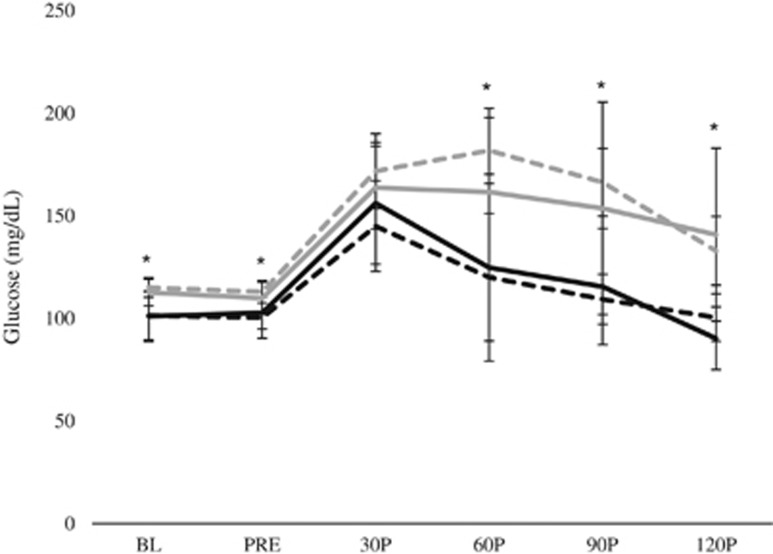
Postprandial glycemic response among individuals with varying levels of glucose tolerance during an OGTT with (D3) and without (D2) the acute BM extract ingestion. Fast_peak_ is represented by a black line (D2) and a dashed black line (D3). Slow_peak_ is represented by a gray line (D2) and a dashed gray line (D3). *Significant difference between groups during D2.

When examining the effects of BM among subjects of differing levels of glucose tolerance (that is, Fast_peak_ and Slow_peak_), no significant group × trial × time interaction was observed for plasma glucose concentrations (*P*=0.168). Similarly, no significant trial × time interaction was observed for either group (Fast_peak_: *P*=0.130; Slow_peak_: *P*=0.429). A significant group × time interaction was noted during D2 (*P*=0.010; Figure 2), wherein Fast_peak_ displayed lower plasma glucose concentrations at BL (95% CI: 86.8–115.9 vs 109.2–120.9; *P*=0.042), PRE (95% CI: 87.9–112.7 vs 106.2–119.7; *P*=0.038), 30P (95% CI: 117.6–172.5 vs 149.1–194.5; *P*=0.070), 60P (95% CI: 81.5–158.6 vs 162.0–201.7; *P*=0.004), 90P (95% CI: 94.2–124.5 vs 145.9–186.8; *P*<0.001) and 120P (95% CI: 86.0–114.8 vs 112.2–153.8; *P*=0.007) compared with Slow_peak_; however, no significant group × time interaction was noted during D3 (*P*=0.208). Furthermore, no significant group × trial interactions were noted at any time point.

No significant between-trial differences were observed among AUC_glu_ (Fast_peak_: *P*=0.529; Slow_peak_: *P*=0.486), Glu_mean_ (Fast_peak_: *P*=0.677; Slow_peak_: *P*=0.559) or Glu_peak_ (Fast_peak_: *P*=0.102; Slow_peak_: *P*=0.523).

### Changes in the postprandial insulin response

When assessing all subjects, no significant trial × time interaction was observed for serum insulin concentrations (*P*=0.307), but significant time effects were noted (*P*=0.033). Serum insulin was significantly elevated from BL (95% CI: 0.5–3.1) at 30P (95% CI: 7.4–75.0; *P*=0.025), 60P (95% CI: 1.7–100.2; *P*=0.048), 90P (95% CI: 5.5–80.2; *P*=0.032) and 120P (95% CI: 7.9–53.7; *P*=0.017). No significant between-trial differences were observed for total AUC_ins_ (*P*=0.836), Ins_mean_ (*P*=0.839) or Ins_peak_ (*P*=0.126).

When assessing the effects of the BM beverage among responders (*n*=5) and non-responders (*n*=5), no significant group × trial × time interaction was observed for serum insulin concentrations (*P*=0.276). Similarly, no significant trial × time interaction was observed for responders (*P*=0.260) or non-responders (*P*=0.695). Additionally, no significant group × time interactions were noted during either trial (D2: *P*=0.177; D3: *P*=0.487); but significant time effects were observed (*P*=0.030). Serum insulin concentrations were elevated from BL (95% CI: 0.4–3.2) at 30P (95% CI: 8.1–74.2; *P*=0.023), 60P (95% CI: 1.8–100.2; *P*=0.047), 90P (95% CI: 6.5–79.1; *P*=0.028) and 120P (95% CI: 9.7–52.0; *P*=0.011). Furthermore, no significant group × trial interactions were noted at any time point.

No significant between-trial differences were observed among AUC_ins_ (responders: *P*=0.324; non-responders: *P*=0.100), Ins_mean_ (responders: *P*=0.355; non-responders: *P*=0.148) or Ins_peak_ (responders: *P*=0.290; non-responders: *P*=0.201).

When examining the effects of BM among subjects of differing levels of glucose tolerance, no significant group × trial × time interaction was observed for serum insulin concentrations (*P*=0.637). Similarly, no significant trial × time interaction was observed for either group (Fast_peak_: *P*=0.421; Slow_peak_: *P*=0.708), and no group × time interaction was observed for either trial (D2: *P*=0.317; D3: *P*=0.305). However, significant time effects were noted (*P*=0.037); serum insulin concentrations were elevated from BL (95% CI: 0.4 to 3.3) at 30P (95% CI: 4.7 to 77.6; *P*=0.035), 60P (95% CI: −0.2 to 102.1; *P*=0.054), 90P (95% CI: 6.5 to 79.1; *P*=0.029) and 120P (95% CI: 10.3 to 51.4; *P*=0.010). No significant group × trial interactions were noted at any time point.

No significant between-trial differences were observed among AUC_ins_ (Fast_peak_: *P*=0.184; Slow_peak_: *P*=0.619), Ins_mean_ (Fast_peak_: *P*=0.272; Slow_peak_: *P*=0.210) or Ins_peak_ (Fast_peak_: *P*=0.233; Slow_peak_: *P*=0.676).

## Discussion

The results of this study indicated that consuming a beverage containing BM extract prior to an OGTT attenuated the postprandial glucose response in 50% of prediabetic participants. The subjects that experienced a reduction in the postprandial glucose response during an OGTT following ingestion of the BM beverage (that is, the responders) displayed lower AUC_glu_ (−7.6% to −17.1%), Glu_mean_ (−6.5% to −17.0%) and Glu_peak_ (−2.1% to −15.6%) during D3. However, when the acute glycemic effects of the beverage were compared across subjects with distinct levels of glucose tolerance (that is, Fast_peak_ vs Slow_peak_), the beverage did not promote an improved glucose response for either group. Interestingly, any observed improvements in the glycemic response occurred independently of an augmented insulin response.

BM has been previously shown to enhance glucose tolerance in diabetics during an OGTT following a single-dose treatment.^6, 9, 10^ However, a number of the investigations demonstrating the efficacy of BM as a hypoglycemic agent have acknowledged the presence of responders and non-responders.^9, 10, 11, 13^ In this sample of prediabetics, 50% of all subjects experienced a significant improvement in their glycemic response to the OGTT following an acute ingestion of the study beverage. This is evidenced by the reduced total AUC_glu_ (−13.2%±3.9%) and mean glucose concentration (−12.2%±4.1%) compared with the control trial (D2). Acute ingestion of the study beverage 30 min prior to the OGTT also attenuated the postprandial increase in blood glucose during D3 compared with D2, indicated by a reduced Glu_peak_ concentration (−10.6%±5.6%) and lower concentrations at 30P (−9.1%±5.2%), 60P (−24.0%±10.3%) and 90P (−20.0%±12.7%). Similarly, Leatherdale *et al.*^6^ observed significant reductions in plasma glucose concentrations and incremental AUC_glu_ among non-insulin-dependent, type 2 diabetics during the initial 90 min of a 120-min OGTT when 50 ml of BM was consumed. Furthermore, others have reported an improved postprandial glycemic response—that is, lowered blood glucose levels—among type 2 diabetics when 100 ml of BM was consumed at least 30 min prior to glucose administration.^9, 10, 19^

In contrast, some studies have reported no significant impact of BM on blood glucose concentrations during OGTT. Specifically, Kasbia *et al.*^11^ observed no significant improvement in the glycemic response during an OGTT when non-diabetic, overweight men consumed 100 mg kg^−1^ of BM juice. In addition, no improvement in plasma glucose levels were reported in healthy rats consuming 0.6 g kg^−1^ of BM extract during an OGTT.^12^ Furthermore, ingestion of 100 ml of BM juice did not improve the glycemic response to a glucose load among insulin-dependent diabetic rats.^19^ Although these opposing conclusions may be due to different forms, dosage and/or preparation of the BM (for example, juice or extract), the inconsistent results may also highlight the individual nature of glucose metabolism. In the present study, subjects displayed two distinct response patterns to the OGTT—those whose highest glucose concentration was observed 30 min after glucose ingestion and those whose highest glucose concentration was observed sometime after the 30-min mark. As the shape of the glucose curve during glucose testing has been suggested to provide metabolic information regarding one's level of glucose tolerance^18, 20^—where greater complexity of the response curve is associated with improved glucose tolerance^21^—this sample appears to represent individuals with slightly impaired (Fast_peak_) and more extensively impaired (Slow_peak_) metabolic condition. Although we were able to classify metabolic responses as Fast_peak_ and Slow_peak_, neither group experienced a significant change in the postprandial glycemic response during an OGTT when the study beverage was consumed 30 min prior to glucose ingestion in the present study.

While some hypothesize that BM acts as an insulin secretagogue *in vivo*^1, 22^ thereby enhancing glucose disposal, others posit that its hypoglycemic effects are due to enhancement of glucose metabolism, augmenting tissue sensitivity to glucose and/or inducing translocation of glucose transporter isoform 4 (GLUT4).^23, 24^ In addition, BM may also inhibit α-glucosidase activity within the small intestine, thus reducing glucose digestion and absorption along the alimentary canal.^13, 23^ Moreover, a primary chemical constituent of BM, polypeptide-p, has demonstrated insulin-mimetic hypoglycemic properties.^2, 22^ Previous research has shown an acute hypoglycemic effect of BM ingestion with^10, 22^ and without^6, 12, 25, 26^ an amplified insulin response, suggesting that the efficacy of BM may be due to the combined effect of multiple mechanisms. In the present study, BM failed to produce a significant insulinogenic effect suggesting that the acute improvement in glucose tolerance may be attributable to extra-pancreatic mechanisms such as increased GLUT4 activity or enhanced glucose uptake in the skeletal muscle and liver. As the aforementioned explanations were beyond the scope of this investigation, future research may consider examining the acute effects of BM ingestion on a molecular level to assess its efficacy with regard to subclinical, prediabetic individuals.

## Conclusion

The present results suggest that the acute ingestion of a beverage containing BM extract may promote an improvement in glucose tolerance during an OGTT among some individuals with impaired glucose regulation. Similar to previous findings, the observed reduction in postprandial glycemia occurred independently of an altered insulin response. Further research is needed to fully elucidate the effects of BM on glucose regulation in prediabetic adults.

## Figures and Tables

**Table 1:** Baseline characteristics of prediabetic subjects

	*Total (*n=*10)*	*Fast*_*peak*_ (n=*5)*	*Slow*_*peak*_ (n=*5)*	*Responders (*n=*5)*	*Non-responders (*n=*5)*
Gender (M/F)	4/6	2/3	2/3	2/3	2/3
Age (years)	60.8 (8.5)	56.3 (8.6)	65.2 (6.1)	64.7 (6.5)	56.8 (8.9)
Body fat (%)	33.1 (12.8)	29.6 (11.3)	36.6 (14.5)	36.1 (14.0)	30.1 (12.3)
BMI (kg m^−2^)	26.2 (5.5)	24.8 (5.2)	27.7 (6.1)	26.4 (4.4)	26.1 (7.1)

Abbreviations: BMI, body mass index; F, female; M, male. Data are reported as mean (s.d.).

**Table 2:** Differences in the glycemic response during an oral glucose tolerance test with (D3) and without (D2) acute bitter melon extract ingestion

	*D2*			*D3*		
	*Total (*n=*10)*	*Responders (*n=*5)*	*Non-responders (*n=*5)*	*Total (*n=*10)*	*Responders (*n=*5)*	*Non-responders (*n=*5)*
BL	108.2 (11.1)	114.0 (6.7)	102.4 (12.2)	106.9 (11.1)	109.9 (10.5)	103.8 (12.0)
PRE	106.6 (10.1)	112.3 (6.9)	101.0 (10.1)	106.3 (8.4)	108.4 (10.2)	104.2 (6.5)
30P	158.4 (23.8)	162.7 (33.9)	154.2 (8.7)	159.9 (24.3)	146.8 (26.2)	173.0 (14.5)
60P	151.0 (40.0)	161.9 (51.7)	140.0 (25.1)	143.2 (45.2)	124.9 (44.8)	161.5 (41.8)
90P	137.8 (33.0)	147.0 (27.0)	128.7 (38.9)	134.6 (44.2)	119.0 (33.6)	150.2 (51.5)
120P	116.7 (21.9)	121.0 (16.6)	112.4 (27.5)	115.6 (40.0)	112.1 (22.6)	119.1 (55.3)

Abbreviations: BL, blood samples collected upon arrival; PRE, blood samples taken immediately prior to glucose intake. 30P, 60P, 90P and 120P, blood samples taken 30, 60, 90 and 120 min after glucose intake, respectively. Data are reported as mean mg dl^−1^ (s.d.).
